# Transcriptome Profiling Reveals Th17-Like Immune Responses Induced in Zebrafish Bath-Vaccinated with a Live Attenuated *Vibrio anguillarum*

**DOI:** 10.1371/journal.pone.0073871

**Published:** 2013-09-04

**Authors:** Hua Zhang, Chao Fei, Haizhen Wu, Minjun Yang, Qin Liu, Qiyao Wang, Yuanxing Zhang

**Affiliations:** State Key Laboratory of Bioreactor Engineering, East China University of Science and Technology, Shanghai, China; Beijing Institute of Microbiology and Epidemiology, China

## Abstract

**Background:**

A candidate vaccine, live attenuated *Vibrio anguillarum* developed in our laboratory could prevent vibriosis of fish resulted from *V. anguillarum* and *V. alginolyticus*. To elucidate the molecular mechanisms underlying the vaccine protection, we used microarray technology to compare the spleen transcriptomes of bath-vaccinated and unvaccinated zebrafish at 28 days post vaccination.

**Principal Findings:**

A total of 2164 genes and transcripts were differentially expressed, accounting for 4.9% of all genes represented on the chip. In addition to iron metabolism related to the innate immunity and the signaling pathways, these differentially expressed genes also involved in the adaptive immunity, mainly including the genes associated with B and T cells activation, proliferation and expansion. Transcription profiles of Th17-related transcription factors, cytokines and cytokine receptors during 35 days post-vaccination implied that Th17 cells be activated in bath-vaccinated zebrafish.

**Conclusion/Significance:**

The transcriptome profiling with microarray revealed the Th17-like immune response to bath-vaccination with the live attenuated *V. anguillarum* in zebrafish.

## Introduction

*Vibrio anguillarum*, a Gram negative, curved rod bacterium, is the causative agent of vibriosis in cultivated fish. The vaccination against *V. anguillarum* is now recognized as a viable strategy for controlling vibriosis. A live attenuated vaccine has been successfully constructed in our laboratory by curing the virulence plasmid pEIB1 which encodes a very efficient iron uptake system mediated by the siderophore anguibactin [1] and deleting the *aroC* gene from the wild-type strain *V. anguillarum*. It shows a remarkable immunogenicity against *V. anguillarum* for *Paralichthys olivaceus*, *Scophthalmus maximus* and zebrafish. Zebrafish vaccinated with the live attenuated vaccine strain show a relative percent survival of 90% [2]. Meanwhile, the live attenuated vaccine also had excellent cross immunoprotection against *Vibrio alginolyticus*, which is the main pathogen causing a large economic lost in the primary cultivated economic fishes, such as *Epinephelus coioides* and *Pseudosciaena crocea*, in southern China (US patent, No: 20080274136, 2010). However, the mechanism of its immune protection induced by bath-vaccination has not yet been fully understood.

The ultimate goal of a vaccine is to develop long-lived immunological protection against a given pathogen depending on long-lived memory cells and effector cells. The memory T-cell compartment consists of both CD4^+^ and CD8^+^ T cells which can rapidly acquire effector functions to kill infected cells and/or secret inflammatory cytokines that inhibit replication of the pathogen [3]. Different classes of microbes elicit lineage-specific responses from the T-cell repertoire. While helper Th1 cells produce large quantity of interferon-γ (IFN-γ) to participate in cellular immunity against intracellular pathogen, Th2 cells produce mainly interleukin-4 (IL-4) to mediate the humoral responses to extracellular pathogen. More recently, a subset of CD4^+^ T cells which were observed to preferentially produce interleukin-17 (IL-17), but not IFN-γ and IL-4, were named Th17 cells. The Th17 cells orchestrate the mucosal defense against pathogen by secreting proinflammatory cytokines IL-17 and IL-22, which stimulate the airway, intestinal and skin epithelia to secrete chemokines and an array of antimicrobial peptides which repel assault from diverse infectious agents [4]–[6]. Th17 response is likely to emerge as an early response to a number of pathogens not handled well by Th1 or Th2-type immunity, which requires robust tissue inflammation to be cleared. Indeed, through the potent induction of chemokines, Th17 cells attract other subsets of T helper cells to sites of infection at later stages of the inflammatory process [7].

Although Th17 cells have not been identified in teleost, it is thought to be an ancient lineage highly conserved in all vertebrates including the jawless lamprey [8], [9]. Zebrafish, for which an enormous amount of information on a genomics scale is available, is a well-studies model for the analysis of host-pathogen interaction during infectious disease [10]. Numerous genes associated with Th17 cells differentiation, function and signaling have been identified in this animal model. Five forms of zebrafish IL-17, the hallmark cytokines of Th17 cells, have been identified [11]. Nuclear RAR-related orphan receptor gamma (RORc), a master regulator of Th17 differentiation, is also conserved in zebrafish [12]. Other cytokines such as IL-21, IL-23 and IL-26 contributing to the Th17 cells differentiation and produced by Th17 cells were discovered in zebrafish too [13], [14]. Very recently, the Th17-like immune responses in fish were reported. It was found that Th17 lineage might be related to the severity of side effects in Atlantic salmon vaccinated with oil-adjuvanted vaccine [15] while a Th17-like immune response was demonstrated to be induced in carp infected with a protozoan parasite [16]. However, Th17-like immune response of teleost induced by bath-vaccination with the live attenuated vaccine has not been reported yet. In this work, the gene expression profiling between control and bath-vaccinated fish at 28 days post-vaccination using Zebrafish Agilent gene expression microarray chips was analyzed. Besides notably activated innate immunity, the expression profiling of the genes related to adaptive immunity responses suggested the maturation of B lymphocytes and the activation of Th17 pathway. In order to demonstrate whether Th17-like immune response be involved in the immune protection against *V. anguillarum*, the differential expression of Th17 pathway-associated genes was investigated in inoculated zebrafish during 35 days post vaccination. The results revealed a Th17-like immune response as well as antibody-mediated immune response mainly contributing to the immunoprotection against *V. anguillarum* in bath-vaccinated zebrafish.

## Materials and Methods

### Ethics Statement

The animal work presented here was approved by the Animal Care Committee, East China University of Science and Technology (approval ID: 2006(272)).

### Fish, Vaccination and Sampling

Six-month-old zebrafish weighting about 0.3 g were obtained from the animal center in our laboratory (Shanghai, China). They were reared in running dechlorinated and aerated water at 24°C on a 12∶12 h light/dark rhythm and fed with commercial feed for aquatic animal twice per day. After 6 days of acclimation, 420 zebrafish were randomly divided into three vaccinated groups and three control groups. *V. anguillarum* MVAV6203 was cultured in high-salt Luria (LB) medium at 30°C for 16 h. The cells were harvested by centrifugation and rinsed twice in 2% saline. The desired number of cells was adjusted to 1×10^8^ CFU/ml with 2% saline. Six groups of 70 zebrafish were immersed in the aerated cell-resuspended saline or 2% saline for 10 min at 24°C. At 1, 7, 14, 21, 28 and 35 days post-vaccination (dpv), 10 zebrafish from each group were euthanized and sacrificed to isolate the spleen tissue. For euthanasia, they were immersed in 300 ng ml^−1^ tricaine methanesulphonate (MS-222, Sigma, USA) for at least 10 min. At 28 days post-vaccination additional pool of spleen tissue of 10 zebrafish from each group was harvested for microarray hybridization.

### RNA Preparation, Microarray Hybridization and Data Analysis

Total RNA was isolated from spleen samples by Trizol (Invitrogen, USA) according to the manufacturer’s instructions. The RNA samples were digested with DNase (Promega, USA) to eliminate genomic DNA contaminant. The quality of RNA samples was assessed using NanoDrop ND-1000 spectrophotometer (Labtech, USA). The integrity of RNA was assessed using standard denaturing agarose gel electrophoresis. From each sample, 1 µg of total RNA was amplified and transcribed into fluorescent cDNA with the manufacturer’s Agilent’s Quick Amp Labeling protocol (version 5.7, Agilent Technologies). The labeled cDNAs were hybridized onto the Whole Zebrafish Genome Oligo Microarray (4×44 K, Agilent Technologies). After having washed the slides, the arrays were scanned by the Agilent Scanner G2505B. Agilent Feature Extraction software (version 10.5.1.1) was used to analyze acquired array images. The software determines feature intensities (including background subtraction), rejects outliers and calculates statistical confidences. Median normalization and subsequent data processing were performed using the GeneSpring GX v11.0 software package (Agilent Technologies). After median normalization of the raw data, genes that at least 4 out of 8 samples have flags in Present (“All Targets Value”) were chosen for further data analysis. Differentially expressed genes with statistical significance were identified through Volcano Plot filtering. To identify differentially expressed genes with statistical significance Fold Change ≥ 1.5 (*p* ≤ 0.05) was used as a cutoff. Pathway analysis and GO enrichment analysis were applied to determine the function of differentially expressed genes in different biological pathways or GO terms. GO analysis was performed with DAVID software tools for Functional Classification and Functional Annotation Clustering (http://david.abcc.ncifcrf.gov/home.jsp) and KEGG pathway database with default parameters. All data set can be downloaded from Gene Expression Omnibus public data base at www.ncbi.nlm.nih.gov/geo/with the GEO accession number GSE39914 (Table S1).

### Real-time Quantitative PCR Analysis

Real-time quantitative PCR was performed using the ABI Prism 7500 Detection System (Applied Biosystems, USA) with SYBR Green (Roche, USA) as the fluorescent detection dye according to the manufacturer’s protocol. Total RNA was isolated from spleen samples by Trizol (Invitrogen) according to the manufacturer’s instructions. The RNA samples were digested with DNase (Promega) to eliminate genomic DNA contaminant. First strand cDNA was synthesized by PrimeScript RT reagent kit (TaKaRa, Dalian, China) with oligo d(T) primer and random 6 mers using DNase digested total RNA as template. Primers were designed using Primer Express 3 software (Applied Biosystems) (Table S2). The thermal cycling profile consisted of an initial denaturation at 95°C for 10 min, followed by 40 cycles of denaturation at 95°C for 15 s an appropriate annealing/extension temperature at 60°C for 60 s. An additional temperature ramping step was utilized to produce melting-curves of the reaction from 60°C to 95°C. For each gene the triplicate fluorescence intensities of the control and treatment groups were measured. The relative expression of each gene was determined by comparative threshold cycle method (2^−ΔΔCt^ method) with house-keeping gene β-actin as reference gene. For each primer a standard curve was generated by analyzing serial dilutions of cDNA to optimize the designed primer. Student’s *t*-test was used to determine whether the detected expression differences were statistically significant (*p*<0.05).

## Results and Discussion

### Global Changes in Gene Expression upon Vaccination

A total of 2164 genes and transcripts were differentially expressed using Fold Change ≥ 1.5 (*p*≤0.05) as a cutoff, accounting for 4.9% of all genes represented on the chip (Table S3). Using the web-based database for annotation, visualization and integrated discovery (DAVID), the gene ontology (GO) analysis of the genes was performed with default settings and the Ensembl Gene IDs was used as input during the process. DAVID had functional annotation for 1436 genes, making up 66.3% of all differently expressed genes. In the “molecular function” and “biological process” categories, “nucleic acid binding” and “cellular metabolic process” were the most abundant GO terms, making up 13.8% and 20.3% of each subcategory, respectively. GO analysis indicated that live attenuated *V. anguillarum* up- and down-regulated genes were involved in cellular metabolic process, response to stress, transcription activator activity (Table 1).

**Table 1:** GO function annotation results of differentially expressed genes.

Term	GO ID	Description	Gene No.	%*	*p* value
BP_2	GO:0044237	Cellular metabolic process	292	20.3	0.0000
BP_2	GO:0009058	Biosynthetic process	140	9.7	0.0015
BP_2	GO:0006807	Nitrogen compound metabolic process	138	9.6	0.0035
BP_2	GO:0048522	Positive regulation of cellular process	25	1.7	0.0114
BP_2	GO:0048518	Positive regulation of biological process	26	1.8	0.0139
BP_2	GO:0055085	Transmembrane transport	44	3.1	0.0141
BP_2	GO:0009893	Positive regulation of metabolic process	13	0.9	0.0148
BP_2	GO:0009056	Catabolic process	46	3.2	0.0198
BP_2	GO:0006996	Organelle organization	39	2.7	0.0328
BP_2	GO:0043170	Macromolecule metabolic process	237	16.5	0.0390
BP_2	GO:0042440	Pigment metabolic process	5	0.3	0.0520
BP_2	GO:0044238	Primary metabolic process	293	20.4	0.0524
BP_2	GO:0043933	Macromolecular complex subunit organization	18	1.2	0.0724
BP_2	GO:0048523	Negative regulation of cellular process	21	1.5	0.0859
BP_2	GO:0006950	Response to stress	36	2.5	0.0889
BP_2	GO:0007163	Establishment or maintenance of cell polarity	4	0.3	0.0980
MF_2	GO:0000166	Nucleotide binding	166	11.6	0.0016
MF_2	GO:0016563	Transcription activator activity	11	0.8	0.0046
MF_2	GO:0003676	Nucleic acid binding	198	13.8	0.0089
MF_2	GO:0008135	Translation factor activity	14	1.0	0.0160
MF_2	GO:0001882	Nucleoside binding	104	7.2	0.0330
MF_2	GO:0048037	Cofactor binding	24	1.7	0.0390
MF_2	GO:0016874	Ligase activity	26	1.8	0.0560
MF_2	GO:0008289	Lipid binding	21	1.5	0.0580
CC_2	GO:0044424	Intracellular part	323	22.5	0.0015
CC_2	GO:0005622	Intracellular	395	27.55	0.0016
CC_2	GO:0031967	Organelle envelope	25	1.75	0.0182
CC_2	GO:0043227	Membrane-bounded organelle	223	15.55	0.0429
CC_2	GO:0043233	Organelle lumen	25	1.75	0.0525
CC_2	GO:0043229	Intracellular organelle	257	17.9	0.0686
CC_2	GO:0044422	Organelle part	87	6.0	0.0816
CC_2	GO:0044446	Intracellular organelle part	87	6.0	0.0816

* the percentage of genes in the specific subcategory from each of the three GO ontologies.

The 2164 genes were mapped to the Kyoto Encyclopedia of Genes and Genomes (KEGG) to identify the biological pathways that were activated in the zebrafish in response to the vaccination. A total of 1766 genes of zebrafish transcriptome were mapped to KEGG and 46 statistically remarkable categories (*p*<0.05) were identified (Table S4), 20 of which were listed in Table 2. Several signaling pathways, including MAPK, insulin, hedgehog, Wnt and RIG-I-like receptor signaling pathways, are significantly modulated.

**Table 2:** Top 20 of Statistically significant KEGG pathways in response to vaccination.

Category	Gene No.	*p* value	FDR
Ribosome	20	0.0000	0.0000
Metabolic pathways	84	0.0000	0.0000
MAPK signaling pathway	31	0.0000	0.0000
Spliceosome	18	0.0000	0.0000
Protein processing inendoplasmic reticulum	21	0.0000	0.0000
RNA transport	18	0.0000	0.0003
Insulin signaling pathway	18	0.0000	0.0006
Hedgehog signaling pathway	10	0.0001	0.0016
Phenylalanine metabolism	5	0.0002	0.0026
Dorso-ventral axis formation	6	0.0004	0.0054
Wnt signaling pathway	17	0.0004	0.0054
Pyrimidine metabolism	12	0.0004	0.0054
Regulation of actin cytoskeleton	20	0.0010	0.0109
Melanogenesis	13	0.0010	0.0109
Protein export	5	0.0013	0.0134
Cytokine-cytokinereceptor interaction	14	0.0018	0.0159
Endocytosis	20	0.0018	0.0159
Tight junction	14	0.0019	0.0159
Purine metabolism	15	0.0021	0.0167
RIG-I-like receptorsignaling pathway	7	0.0044	0.0336

**Figure 1:**
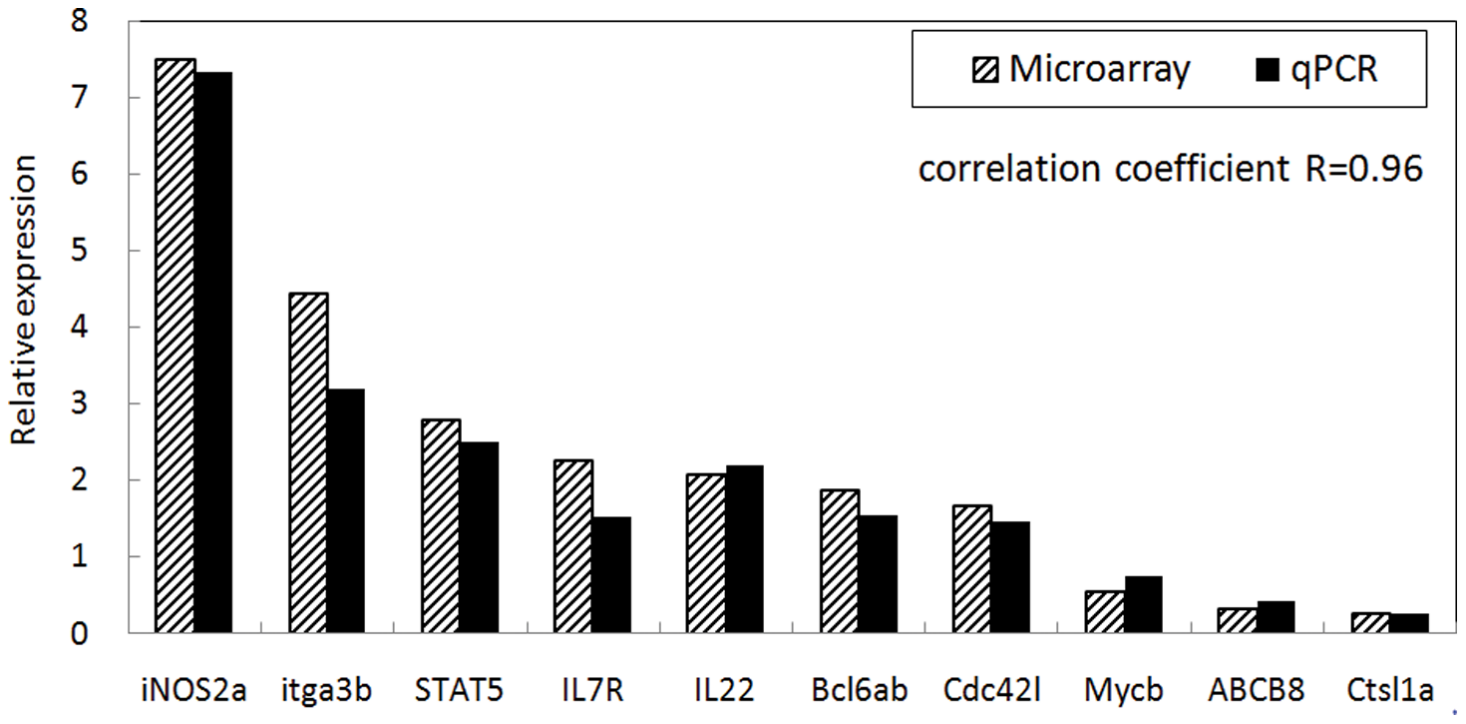
Validation of relative expression between microarray data and RT-qPCR results at 28 days post vaccination. iNOS2a: inducible nitric oxide synthase 2, itga3b: integrin alpha 3b, STAT5: Signal Transducer and Activator of Transcription 5, IL7R: interleukin 7 receptor, IL22: interleukine 22, Bcl6ab: B-cell CLL/lymphoma 6a, Cdc42l: cell division cycle 42 like, Mycb: myelocytomatosis oncogene b, ABCB8: ATP-binding cassette sub-family B member 8, Cts1a: cathepsin L 1 a.

To validate the differentially expressed genes identified by microarray, 10 differentially expressed genes such as inducible nitric oxide synthase 2 (iNOS2a), itga3b, STAT5, IL7R, IL22, Bcl6ab, Cdc42l, Mycb, ABCB8, and Ctsl1a were selected for real-time qPCR analysis. The expressions of all examined genes matched the microarray data (Figure 1).

Many genes associated with the innate immunity were modulated in vaccinated zebrafish with the live attenuated *V. anguillarum* (Table 3). A receptor of inflammatory mediator, Leukotriene B4 receptor, was over-expressed significantly (fold = 9.19) while a scavenger receptor named mannose receptor C type 1 was up-regulated (fold = 6.33). Metalloproteins (MMPs) regulate the cell matrix composition and are evaluated as markers of inflammation [17]. Of them, MMP-9 is important for leukocyte migration and inflammation owing to its ability to degrade basement membranes and components of the extra cellular matrix [18]. MMP-13 has a key role in the MMP activation cascade and also contributes to wound repair [19]. The expressions of the two metalloproteins, MMP9 and MMP13, were up-regulated for 6.12 fold and 2.05 fold, respectively.

**Table 3:** List of differentially expressed genes related to innate immunity.

Accession Number	Gene Name	*p* value	Fold	Description
**Innate immunity**				
XM_002662721	LTB4R	0.0057	9.19	Leukotriene B4 receptor 1-like
NM_001104937	iNOS2a	0.0310	7.50	Nitric oxide synthase 2a, inducible
XR_084418.2	MRC1	0.0004	6.33	Mannose receptor C type 1-like (LOC100329625)
NM_201503	MMP13a	0.0481	6.12	Matrix metalloproteinase 13a
XM_683237	Prf1	0.0088	5.69	Novel protein similar to mouse and rat perforin 1 (Pore forming protein) (Prf1) Fragment
NM_205554	atp6v0cb	0.0109	2.33	ATPase, H^+^ transporting, lysosomal, V0 subunit c, b
NM_173255	atp6v0ca	0.0248	2.27	ATPase, H^+^ transporting, lysosomal, V0 subunit c, a
NM_213123	MMP9	0.0192	2.05	Matrix metalloproteinase 9
NM_199865	Cdc42l	0.0206	1.66	Cell division cycle 42, like
NM_205554	atp6v0cb	0.0078	1.62	ATPase, H^+^ transporting, lysosomal, V0 subunit c, b
**Iron metabolism**				
NM_001017734	Steap4	0.0156	3.10	STEAP family member 4
NM_001128234	SLC30a10	0.0365	2.75	Solute carrier family 30 (zinc transporter), member 10
BC092881	ISCU	0.0303	2.45	Iron-sulfur cluster scaffold homolog
NM_001103139	Hmox2a	0.0445	2.32	Heme oxygenase (decycling) 2
NM_001076602	SLC25a38a	0.0370	2.27	Solute carrier family 25, member 38
NM_001040370	SLC11a2	0.0007	2.22	Solute carrier family 11 (proton-coupled divalent metal iron transporters),member 2
NM_200486	SLC25a39	0.0350	2.02	Solute carrier family 25, member 39
NM_201192	PCBP2	0.0212	1.91	Poly(rC) binding protein 2
XM_001341755	IREB2	0.0009	1.64	Iron-responsive element binding protein 2
NM_001017544	ABCB8	0.0059	−3.09	ATP-binding cassette, sub-family B (MDR/TAP), member 8
NM_199659	cul1a	0.0387	−4.77	cullin 1a
NM_001076601	Blvra	0.0460	−6.08	Biliverdinreductase A
NM_001076662	SFXN4	0.0030	−8.34	Sideroflexin 4
NM_001045438	TMEM14c	0.0039	−10.68	Transmembrane protein 14C
NM_213021	Glrx5	0.0204	−11.97	Glutaredoxin 5 homolog (*S. cerevisiae*)
BC107969	TfR2	0.0051	−12.93	Transferrin receptor 2
NM_201306	LRPAP1	0.0141	−13.79	Low density lipoprotein receptor-related protein associated protein 1

**Figure 2:**
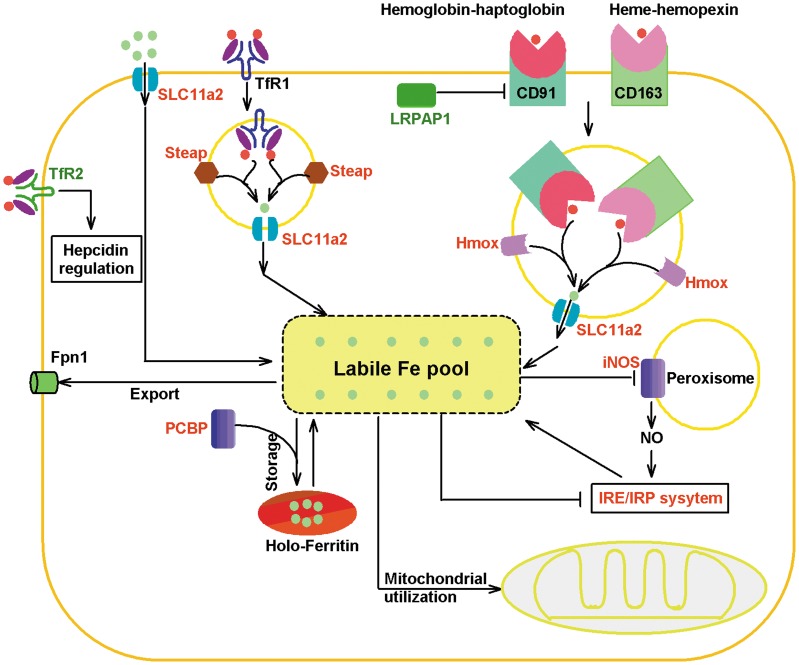
Iron uptake and metabolism were enhanced in the bath-vaccinated zebrafish. Genes related to putative iron metabolism of intracellular iron and competition for extracellular iron were differentially modulated after vaccination with the live attenuated *V. anguillarum*. Red: up-regulated, Green: down-regulated, Black: not found to be modulated.

Additionally, a number of genes related to iron metabolism were differentially expressed in the spleen of zebrafish at 28 days post bath-vaccination. The nature resistance associated macrophage protein 2 (Nramp2, also known as SLC11a2) is a transporter associated with export of iron from phagosomes [20]. Its up-regulated expression (fold = 2.22) might increase the iron uptake from transferrin and low-molecular-weight iron complexes. *In vitro* uptake of hemopexin-heme complex mediated by CD91 could be inhibited by LRPAP1 (low density lipoprotein receptor-related protein associated protein 1) [21]. The expression of LRPAP1 was significantly suppressed (fold = −13.79). The expression of IREB2 (iron responsive element binding protein 2) which plays a central role in iron metabolism was slightly increased (fold = 1.64). IREB2 can sense cytosolic iron levels and posttranscriptionally regulate iron metabolism genes including transferrin receptor 1 (TfR1) and ferritin H and L subunits, by binding to iron-responsive elements (IREs) within target transcripts. Its up-regulated expression implied the more iron uptake. Although the expression of transferrin receptor 2 (Tfr2) which might play a role in cellular iron uptake through binding and internalizing a carrier protein transferrin (Tf) decreased considerably, its substantially lower affinity to Tf was identified and its expression was independent on cellular status of iron in contrast to Tfr1 [22]. Its decreased expression might be related to other function. Additionally, NO is a critical regulator of cellular iron homeostasis via activation of IRP (iron regulatory protein) binding to IRE (iron responsive element). The expression of iNOS2a, which could produce important antimicrobial effectors namely nitric oxide (NO) [23], was highly up-regulated (fold = 7.50). As a ferritin iron chaperone, poly(rC) binding protein (PCBP) could deliver iron to ferritin. Its up-regulated expression implied that excess iron was stored as the form of ferritin. Taken together, the genes expression changes indicated that iron uptake was increased after bath-vaccination (Figure 2).

Meanwhile, the expressions of numerous genes relevant to adaptive immunity responses were regulated, including cytokines, receptors and other proteins involved in the antigen presentation and the T-cell and B cell activation (Table 4). Expression of some immunologically significant genes increased, including TCRVR, CD28, CREB, MHCII, CD276 (B7) and CD40, which could provide the activating signal and co-stimulation signal to naive T cell. From these evidences, helper T lymphocytes were activated by bath-vaccination of the live attenuated *V. anguillarum*. Besides, The expressions of four transcription factors Bcl6, Prdm1a, Nfxl1, and STAT6 which were found to play important roles in B cells activation and differentiation and another transcription factor RBPJ (recombination signal binding protein for immunoglobulin kappa J region) which was involved in cell fate determination of margin zone B cell [24] were all elevated. Especially, Bcl6 is a master factor of B cell differentiation. These results suggest that the differentiation and maturation of B lymphocytes were induced. In our laboratory, unambiguous specific antibody response was detected in serum of vaccinated zebrafish during 28 days post vaccination [2]. Significantly enhanced specific antibody and changes in gene expression related to B cell differentiation at 28 days post bath-vaccination of live attenuated *V. anguillarum* confirmed responses of humoral immunity.

**Table 4:** List of differentially expressed genes related to adaptive immunity.

Accession number	Gene name	*p* value		Fold	Description
**Cytokines and cytokine receptors**					
NM_001043334	EpoR	0.0025	9.24		Erythropoietin receptor, involved with erythroidand lymphoid differentiation
NM_001113595	CCL20	0.0119	3.27		Chemokine (C-C motif) ligand 20
BC083364	XCR1a	0.0043	3.16		Chemokine (C motif) receptor 1
NM_001037683	TGFBR1a	0.0367	3.12		Transforming growth factor, beta receptor 1 a
BC098597	IL1β	0.0395	2.62		Interleukin 1, beta
NM_194386	TGFb3	0.0326	2.52		Transforming growth factor, beta 3
NM_001083868	crfb4	0.0098	2.35		Cytokine receptor family member b4
NM_001113507	IL7R	0.0109	2.24		Interleukin 7 receptor
BC163192	IL22	0.0228	2.08		Interleukin 22
NM_207640	ifnphi1	0.0467	1.94		Interferon phi 1 (IFNΦ), mRNA
NM_205762	traf4a	0.0449	1.58		TNF receptor-associated factor 4a
NM_001113625	trap1	0.0165	1.55		TNF receptor-associated protein 1
**Antigen present, T-cell and B cell activation**					
AF246168	TCRAV	0.0061		5.96	Isolate G5209 T-cell receptor alpha variable region mRNA, partial cds.
NM_001190309	itga3b	0.0181		4.44	Integrin, alpha 3b
NM_212634	alcamb	0.0073		3.38	Activated leukocyte cell adhesion molecule b
XM_002666544	Nfxl1	0.0003		3.32	Nuclear transcription factor, X-box binding-like 1 (Named Xbp1 in human)
XM_001923447	CD28	0.0236		3.23	T-cell-specific surface glycoprotein CD28-like (LOC100151365)
NM_194387	STAT5.1	0.0112		3.20	Signal transducer and activator of transcription 5.1
DN898368	itgb7	0.0016		3.13	Integrin, beta 7
NM_001040369	tnfrsf9a	0.0465		3.13	TNF receptor superfamily, member 9a
AY841759	Prdm1a	0.0248		3.07	PR domain containing 1a, with ZNF domain (homolog to Blimp of human)
NM_001082997	CD40	0.0037		2.57	CD40 molecule, TNF receptor superfamily member 5
NM_198878	rbpja	0.0033		2.47	Recombination signal binding protein for immunoglobulin kappa J region a
NM_200909	creb1a	0.0042		2.31	cAMP responsive element binding protein 1a
NM_001007167	MHC II	0.0483		1.88	Major histocompatibility complex class II (MHC II) DAB gene
NM_200366	STAT6	0.0162		1.87	signal transducer and activator of transcription 6, interleukin-4 induced
NM_001100074	Bcl6ab	0.0287		1.86	B-cell CLL/lymphoma 6a, genome duplicate b (similar to Bcl-6 of human)
NM_001110403	hspa8	0.0149		1.81	Heat shock protein 8
NM_001080622	CD276	0.0421		1.75	CD276 molecule
NM_214716	hspa4a	0.0492		1.70	Heat shock protein 4a
NM_200172	Mycb	0.0217		−1.88	Myelocytomatosis oncogene b
NM_131198	Ctsl1b	0.0120		−2.05	Cathepsin L b, hatching gland gene 1
NM_001045076	hspa14	0.0310		−2.07	Heat shock protein 14
NM_001089476	hsf5	0.0168		−2.06	Heat shock transcription factor family member 5
BC090693	FNYBb	0.0455		−2.38	Nuclear transcription factor Y, beta b
BC063995	mhc1ze	0.0477		−3.25	Major histocompatibility complex class I ZE gene
NM_212584	Ctsl1a	0.0435		−3.80	Cathepsin L 1a
NM_200075	hspbp1	0.0307		−4.90	HSPA (heat shock 70 kDa) binding protein, cytoplasmic cochaperone 1
NM_213522	igbp1	0.0051		−4.95	Immunoglobulin (CD79A) binding protein 1
XM_001343036	Ciita	0.0413		−5.61	Class II, major histocompatibility complex, transactivator
NM_198210	hsp90b1	0.0277		−8.64	Heat shock protein 90, beta (grp94), member 1

### Cytokines, Membrane Receptors and Transcription Factors Associated with Th17 Cell

As the functions of those genes related to adaptive immunity were further explored, we found that several genes associated with Th17 differentiation and amplification were significantly modulated. In contrast to Th1 and Th2 cells which depend on their respective effector cytokines (IFN-γ and IL-4) for differentiation, Th17 differentiation is initiated by the combined action of IL-6, TGF-β [25], [26] and IL-1β [27]. The genes encoding IL-1β, TGF-β and TGF-β receptor in vaccinated zebrafish were up-regulated. Furthermore, the mannose receptor (MRC1) was overexpressed by 6.33 fold (*p* = 4.4×10^−4^). Although its immunological role is still unknown, a possible clue is via a role in inducing Th17 response during infection [28].

The expression of gene encoding IL22, one of cytokines secreted by Th17 effector, was elevated by 2.08 fold. Furthermore, Cua *et al* recently indicated that IL-23 might play an important role in the terminal differentiation of Th17, potentially through its effect on re-expression of IL7R on Th17 cells [29]. Activated Th17 cells and other effector T cells showed surface expression of interleukin 7 receptor (IL7R). IL7R is essential in survival and development of cells differentiating to T cells as well as in mature T cells [30]. In addition, IL7R signaling combined with activation of STAT5 appears to play an essential, although not sufficient, role for development of memory CD4^+^ T cell [31], [32]. In this work, significant up-regulations of IL23R, IL7R and STAT5 expressions were found in 28 days post bath-vaccinated zebrafish.

**Figure 3:**
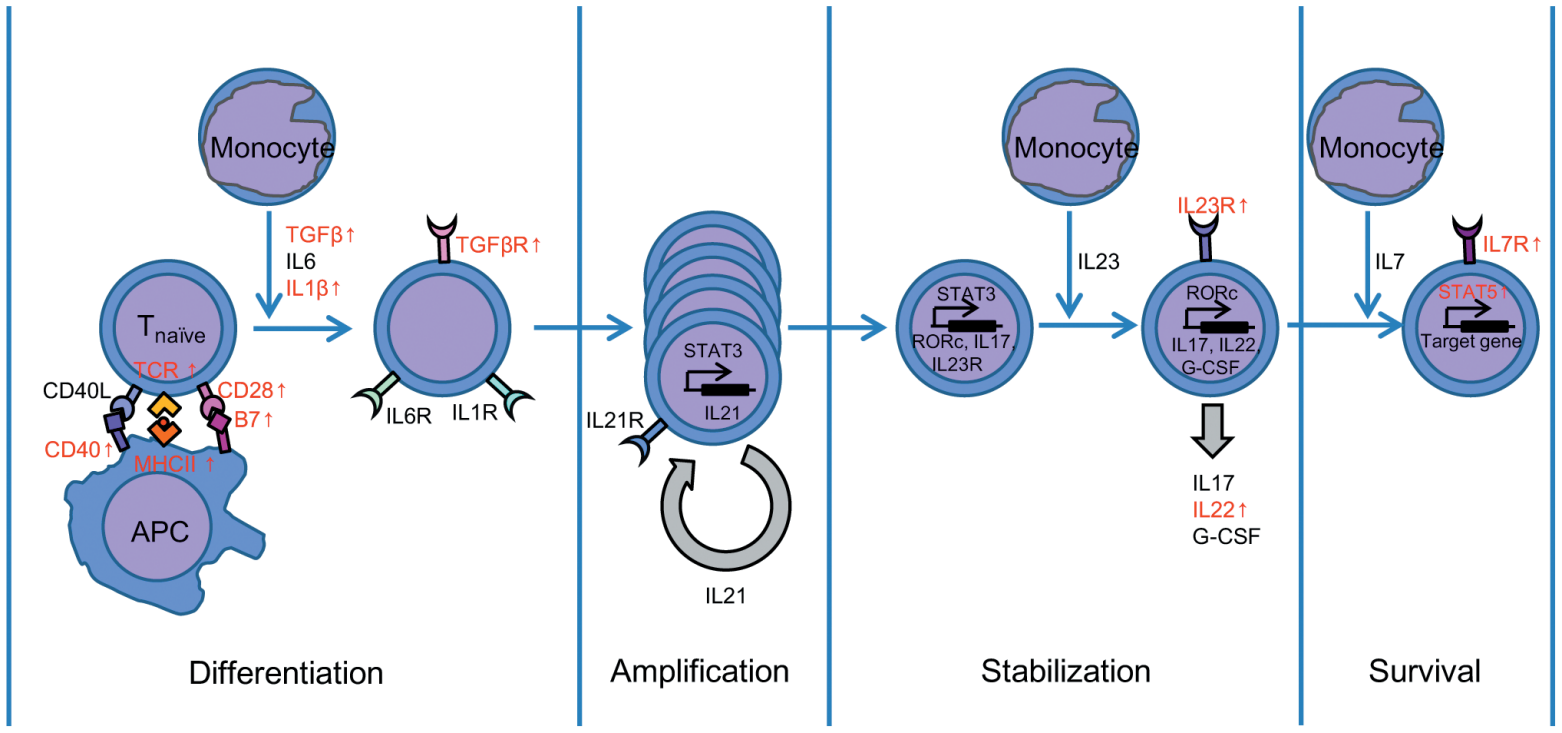
Putative differentiation pathway of Th17 cells in zebrafish. The genes related to Th17 cells differentiation and proliferation were modulated after vaccination with the live attenuated *V. anguillarum*. Red: up-regulated, Black: not found to be modulated.

IL-17 strongly recruits and activates neutrophils [33] and stimulates the release of a variety of mediators of inflammation including matrix metalloproteinases such as MMP9 [34] and MMP13 [35] which facilitate neutrophil infiltration. CCL20, a small cytokine belonging to the CC chemokine family, is strongly and specifically chemotactic for Th17 cells by its CCR6 chemokine receptor and is secreted by Th17 cells [36]. In this work, the gene expressions of all three mediators CCL20, MPP9, and MMP13 were up-regulated. Accordingly, it could be believed that the Th17-like immune response was activated in bath-vaccinated zebrafish with the live attenuated *V. anguillarum* (Figure 3).

Th17 cells preferentially migrate to the intestine and mucosa-associated lymphoid tissues [37] in mammal. Integrin β7, a subunit of integrin receptor and with the alpha 4 subunit, forms a specific lymphocytes receptor which is important for effectors and memory lymphocytes migration into gut [38], [39]. In addition, BLT1, the receptor for the potent lipid chemoattractant leukotriene B4 (LBT4), can mediate the recruitment of neutrophils and effector T cells [40], [41]. The expression of both receptors were up-regulated at 28 dpv post bath-vaccination. Accordingly, It is suggested that bath vaccination by the live attenuated *V. anguillarum* might enhance leukocytes chemotaxis in spleen.

### Temporal Expression Profiling of Th17-related Genes

**Figure 4:**
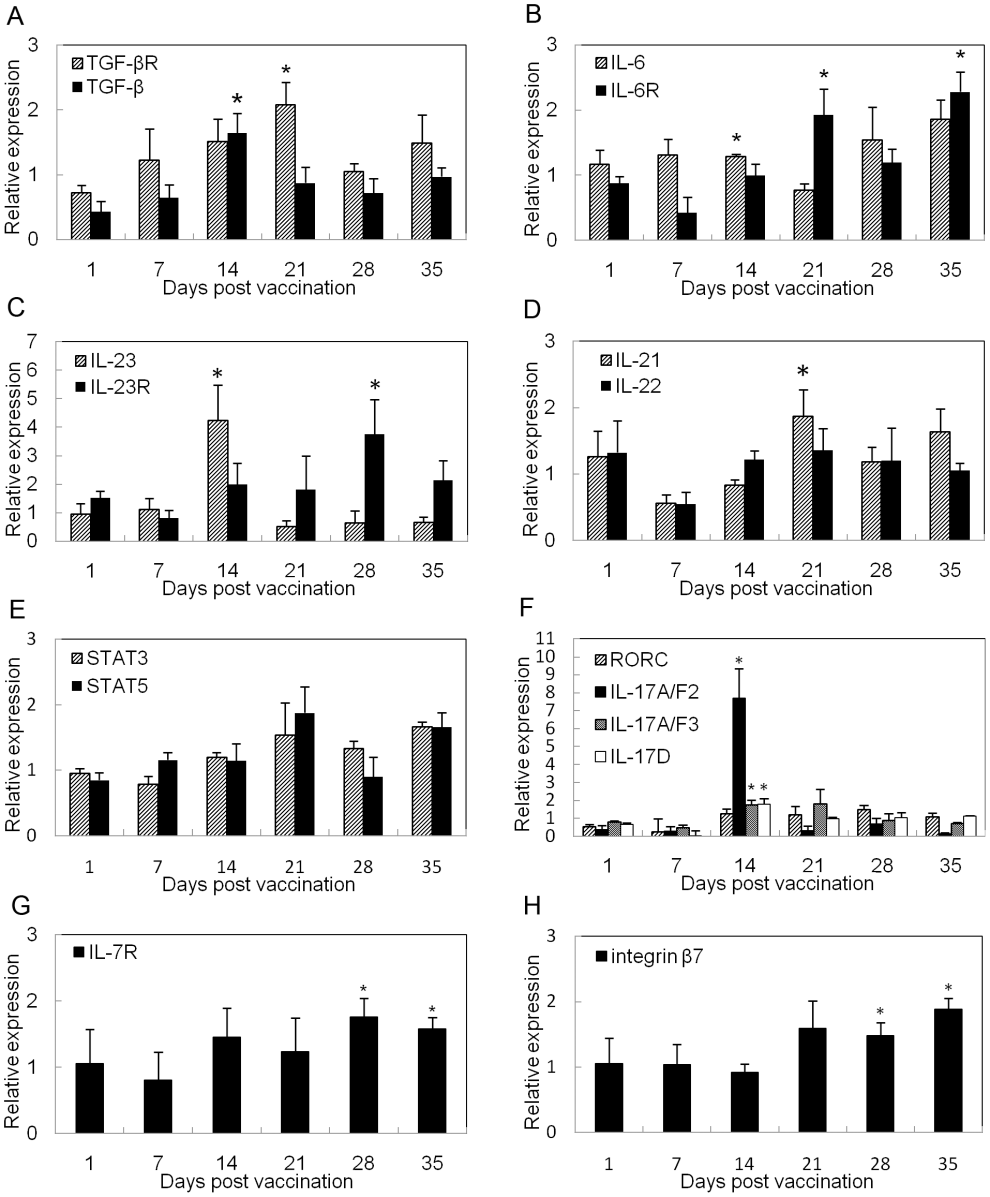
Temporal expression profiling of Th17-related genes and integrin β7 during 35 days post bath-vaccination. The mRNA level of each gene was normalized to that of β-actin. Each bar represents the mean of three biological replicates (3 pools) and error bars represent standard deviation. (^*^ *p*<0.05).

Microarray analysis showed the signs of adaptive T cell immunity. Several up-regulated cytokines (TGF-β, IL-1β, IL-22), surface receptors (TGF-βR, IL-23R and IL-7R), and transcription factor STAT5 illustrated Th17 differentiation and expansion. To confirm the activation of Th17, a set of Th17-related markers were included in a temporal expression analysis during 35 days post bath-vaccination with the live attenuated *V. anguillarum*. Of the 16 genes measured, the expressions of 14 genes were significantly changed in zebrafish after vaccination as compared to control group (*p*<0.05). Among them, the expressions of 6 genes were significantly increased at 14 dpv (*p*<0.05), including TGF-β, IL-6, IL-23p19, IL-17A/F2, IL-17A/F3 and IL-17D (Figure 4, A, B, C, F). While TGF-β and IL-6 expressions were slightly, but significantly, elevated, IL-23p19 expression increased with a high peak (4-fold) at 14 dpv. The expressions of the cytokine receptors of TGF-β and IL-23 were up-regulated at 14 dpv. All three cytokines TGF-β, IL-6 and IL-23p19 play important roles in the initiation and maintenance of Th17 differentiation. IL-23 expands and stabilizes Th17 cells to produce their effector cytokines [42]. Particularly, the expression of IL-17A/F2 was highly up-regulated to the peak of over 7-fold at 14 dpv with a gently up-regulated expression of its transcription factor RORc. The expressions of two other transcription factors, STAT3 and STAT5, gradually increased after 7 dpv and reached a peak at 28 dpv (Figure 4, E). Two other cytokines IL-21 and IL-22, mainly secreted by Th17 effector, were elevated with a peak at 21 dpv and 28 dpv, respectively (Figure 4, D). Taken together, these results suggest that Th17 effector cell be activated between 14 dpv to 21dpv. The significantly up-regulated expression of the IL7R and integrin β7 at 28 days and 35 days post vaccination (Figure 4, G, H) imply the exist of immunological memory and mucosal-related immunity.

The expression profiles supported the Th17-like immune response to the live attenuated *V. anguillarum*. In mammals, studies of infectious disease highlight the critical role of Th17 response in host defense against extracellular pathogens, particularly Gram-negative bacteria that colonize mucosal surfaces. Mice infected by *Klebsiella pneumonia* [43], [44], *Bordetella pertussis* [45], or *Streptococcus pneumonia*, for example, mount a Th17 response and disruption of IL-17 signaling increase susceptibility in these model. The activation of Th17 lineage by pertusis toxin and pneumococcal antigen is necessary to confer full protection against subsequent infection [46]. Similar to *K. pneumonia* and *B. pertussis*, *V. anguillarum* is a Gram-negative, extracellular pathogenic bacterium. Following these reality and results, we assume that Th17 lineage provide protection against further infection in the bath-vaccinated zebrafish, although more evidences need to be established.

### Signaling Pathway and Adaptive Immunity

**Figure 5:**
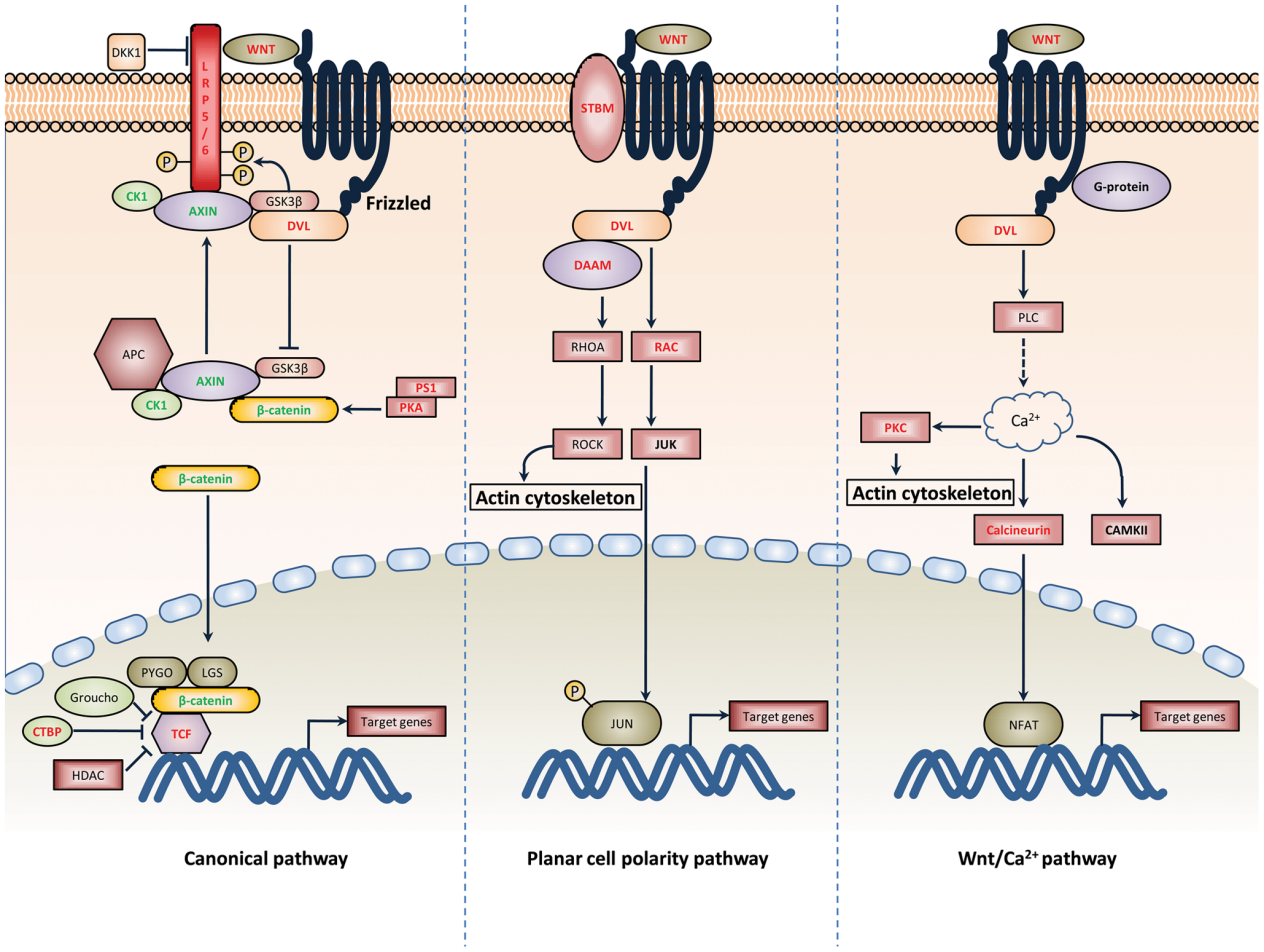
Effects of vaccination with the live attenuated *V. anguillarum* on Wnt signaling pathway. The genes related to Wnt signaling pathway were modulated after vaccination with the live attenuated *V. anguillarum*. Red: up-regulated, Green: down-regulated, Black: not found to be modulated. WNT: wingless-type MMTV integration site family, DKK1: Dickkopf homologue 1, LRP5/6: LDL-receptor-related protein 5/6, CK1: casein kinase, GSK3β: glycogen synthase kinase 3β, AXIN: axis inhibition protein, DVL: mammalian homologue of *Drosophila* dishevelled, APC : adenomatous polyposis coli, PKA: cAMP-dependent protein kinase catalytic subunit alpha, PS-1: presenilin 1, PYGO: legless CREB binding protein, LGS: pygopus CREB binding protein, TCF: T-cell factor, GRG:Groucho, CTBP: C-terminal binding protein, HDAC: histone deacetylases, STBM: VANGL planar cell polarity protein 2, DAAM: dishevelled associated activator of morphogenesis, RHOA: ras homolog family member A, RAC: ras-related C3 botulinum toxin substrate, ROCK: Rho-associated coiled-coil containing protein kinase, JNK: Jun N-terminal kinase, JUN: jun proto-oncogene, PLC: phospholipase C, PKC: protein kinase C, CAMKII: calcium calmodulin mediated kinase II, NFAT: nuclear factor of activated T cells.

Several signaling pathway, such as Wnt and hedgehog (Hh) were significantly modulated at 28 days after bath-vaccination. During embryonic development complex but delicate interactions of these pathways are crucial for stem cell maintenance, body patterning, cell fate determination and organogenesis. Recent studies indicated that the signaling pathway was also associated with lymphocytes development, activation and differentiation. Among them, Wnt-signaling plays a prominent role in the immune system for regulating effector T-cell development, regulatory T-cell activation and dendritic-cell maturation [47]. The expressions of most genes involved in canonical and non-canonical Wnt-signaling pathways were affected in 28 days post vaccination of the zebrafish with the live attenuated *V. anguillarum* (Figure 5). Two reports demonstrated the critical requirements of the Wnt-β-catenin pathway for Th2 differentiation [48], [49]. Although the TCF-1/β-catenin complex positively regulates Th2 initiation and further differentiation, TCF-1 represses alternative Th1 and Th17 fates in activated CD4^+^ T cells [50]. Given the timing for microarray hybridization is at 28 dpv, it is suggested that at this time point Th17-mediated immune response decrease and Th2-meiated B cell maturation are induced. Emerging trends also highlight the capacity of Th17 cells to bridge the gap between innate and adaptive immunity and attract other subset of T helper cell to sites of infection at later stages of the inflammatory process [51]. This deduction is consistent with our results that peak of Th17-mediated immune response appears at 14 dpv and specific antibody response at 28 dpv. Furthermore, Wnt/Ca^2+^ signaling activates NFAT, NF-κB and Bcl6 and up-regulates CD40 expression. NFAT may intensify Wnt/Ca^2+^ signaling by activating NF-κB and Bcl6 for protection of B cells [52]. Hedgehog signaling modulates mature T cell functions through the regulation of cell cycle progression [53]–[55]. Our observation indicated that adaptive immunity induced by the live attenuated *V. anguillarum* are mainly mediated by Th17 cells and B cells.

## Conclusions

The analysis of the transcriptome and gene expression in the live attenuated *V. anguillarum*-vaccinated zebrafish revealed the changes of genes involved in both innate and adaptive immunity. Adaptive immunity associated with Th17-like immune response and antibody response can account for the high level RPS of bath-vaccinated zebrafish. Activation of Th17 pathway implies that bath-vaccination of the live attenuated *V. anguillarum* evokes mucosa immune response which plays pivotal role in orchestrating the mucosal barrier against pathogen. These findings provided valuable leads for further investigation into the mucosal immune response induced by immersion route. The modification of immunity factors and anti-infection at the mucosal site of entry may be a focus in the next work.

## Supporting Information

Table S1 The matrix table deposited at GEO.
(XLS)

Table S2 Primers used for real-time quantitative PCR analysis.
(XLS)

Table S3 Complete list of differentially expressed genes.
(XLS)

Table S4 Complete list of KEGG pathways of differentially expressed genes.
(XLS)

## References

[1] Wu H, Ma Y, Zhang Y, Zhang H (2004) Complete sequence of virulence plasmid pEIB1 from the marine fish pathogen *Vibrio anguillarum* strain MVM425 and location of its replication region. J Appl Microbiol 97: 1021–1028. 15479418 10.1111/j.1365-2672.2004.02387.x

[2] Zhang ZH, Wu HZ, Xiao JF, Wang QY, Liu Q, et al (2012) Immune responses of zebrafish (*Danio rerio*) induced by bath-vaccination with a live attenuated *Vibrio anguillarum* vaccine candidate. Fish Shellfish Immunol 33: 36–41. 22507197 10.1016/j.fsi.2012.03.031

[3] Kaech SM, Wherry EJ, Ahmed R (2002) Effector and memory T-cell differentiation: implications for vaccine development. Nat Rev Immunol 2: 251–262. 12001996 10.1038/nri778

[4] Aujla SJ, Chan YR, Zheng M, Fei M, Askew DJ, et al (2008) IL-22 mediates mucosal host defense against Gram-negative bacterial pneumonia. Nat Med 14: 275–281. 18264110 10.1038/nm1710 PMC2901867

[5] Zheng Y, Valdez PA, Danilenko DM, Hu Y, Sa SM, et al (2008) Interleukin-22 mediates early host defense against attaching and effacing bacterial pathogens. Nat Med 14: 282–289. 18264109 10.1038/nm1720

[6] Liang SC, Tan XY, Luxenberg DP, Karim R, Dunussi-Joannopoulos K, et al (2006) Interleukin (IL)-22 and IL-17 are coexpressed by Th17 cells and cooperatively enhance expression of antimicrobial peptides. J Exp Med 203: 2271–2279. 16982811 10.1084/jem.20061308 PMC2118116

[7] Khader SA, Bell GK, Pearl JE, Fountain JJ, Rangel-moreno J, et al (2007) IL-23 and IL-17 in the establishment of protective pulmonary CD4^+^ T cell responses after vaccination and during *Mycobacterium tuberculosis* challenge.Nat Immunol. 8: 369–377. 10.1038/ni1449 17351619

[8] Savan R, Sakai M (2006) Genomics of fish cytokines. Comp Biochem Phys D 1: 89–101. 10.1016/j.cbd.2005.08.005 20483237

[9] Tsutsui S, Nakamura O, Watanabe T (2007) Lamprey (*Lethenteron japonicum*) IL-17 upregulated by LPS-stimulation in the skin cells. Immuno genetics 59: 873–882. 10.1007/s00251-007-0254-2 17924104

[10] Phelps HA, Neely MN (2005) Evolution of the zebrafish model: from development to immunity and infectious disease. Zebrafish 2: 87–103. 18248169 10.1089/zeb.2005.2.87

[11] Gunimaladevi I, Savan R, Sakai M (2006) Identification, cloning and characterization of interleukin-17 and its family from zebrafish. Fish Shellfish Immunol 21: 393–403. 16677828 10.1016/j.fsi.2006.01.004

[12] Flores MV, Hall C, Jury A, Crosier K, Crosier P (2007) The zebrafish retinoid-related orphan receptor (*ror*) gene family.Gene Expr Patterns. 7: 535–543. 10.1016/j.modgep.2007.02.001 17374568

[13] Holt A, Mitra S, van der Sar AM, Alnabulsi A, Secombes CJ, et al (2011) Discovery of zebrafish (*Danio rerio*) interleukin-23 alpha (IL-23a) chain, a subunit important for the formation of IL-23, a cytokine involved in the development of Th17 cells and inflammation. Mol Immunol 48: 981–991. 21324528 10.1016/j.molimm.2010.12.012

[14] Igawa D, Sakai M, Savan R (2006) An unexpected discovery of two interferon gamma-like genes along with interleukin (IL)-22 and-26 from teleost: IL-22 and-26 genes have been described for the first time outside mammals. Mol Immunol 43: 999–1009. 16005068 10.1016/j.molimm.2005.05.009

[15] Stephen M, Glenn AC, Inderjit SM, Ben K, Øystein E (2010) High gene expression of inflammatory markers and IL-17A correlates with severity of injection site reactions of Atlantic salmon vaccinated with oil-adjuvanted vaccines. BMC Genomics 11: 336. 20507624 10.1186/1471-2164-11-336 PMC2996971

[16] Ribeiro CM, Pontes MJ, Bird S, Chadzinska M, Scheer M, et al (2010) Trypanosomiasis- induced Th17-like immune responses in carp. PLoS ONE 5: e13012. 20885956 10.1371/journal.pone.0013012 PMC2946394

[17] Johnson LL, Dyer R, Hupe DJ (1998) Matrix metalloproteinases. Curr Opin Chem Biol 2: 466–471. 9736919 10.1016/s1367-5931(98)80122-1

[18] Dubois B, Masure S, Hurtenbach U, Paemen L, Heremans H, et al (1999) Resistance of young gelatinase B-deficient mice to experimental autoimmune encephalomyelitis and necrotizing tail lesions. J Clin Invest 104: 1507–1515. 10587514 10.1172/JCI6886 PMC409857

[19] Leeman MF, Curran S, Murray GI (2002) The structure, regulation, and function of human matrix metalloproteinase13. Crit Rev Biochem Mol Biol 37: 149–166. 12139441 10.1080/10409230290771483

[20] WardropS RichardsonD (2000) Interferon-γ and lipopolysaccharide regulate the expression of Nramp2 and increase the uptake of iron from low relative molecular mass complexes by macrophages. Eur J Biochem 267: 6586–6593. 11054110 10.1046/j.1432-1327.2000.01752.x

[21] Hvidberg V, Maniecki MB, Jacobsen C, Højrup P, Møller HJ, et al (2005) Identification of the receptor scavenging hemopexin-heme complexes. Blood 106: 2572–2579. 15947085 10.1182/blood-2005-03-1185

[22] Kawabata H, Germain RS, Vuong PT, Nakamaki T, Said JW, et al (2000) Transferrin receptor 2-α supports cell growth both in iron-chelated cultured cells and in vivo. Journal of Biological Chemistry 275 (22): 16618–16625. 10.1074/jbc.M908846199 10748106

[23] Bogdan C (2001) Nitric oxide and the immune response. Nat Immunol 2: 907–916. 11577346 10.1038/ni1001-907

[24] Tanigaki K, Han H, Yamamoto N, Tashiro K, Ikegawa M, et al (2002) Notch-RBP-J signaling is involved in cell fate determination of marginal zone B cells. Nat Immunol 3: 443–450. 11967543 10.1038/ni793

[25] Bettelli E, Carrier Y, Gao WD, Korn T, Strom TB, et al (2006) Reciprocal developmental pathways for the generation of pathogenic effector T_H_17 and regulatory T cells. Nature 441: 235–238. 16648838 10.1038/nature04753

[26] Mangan PR, Harrington LE, O’Quinn DB, Helms WS, Bullard DC, et al (2006) Trans- forming growth factor-β induces development of the T_H_17 lineage. Nature 441: 231–234. 16648837 10.1038/nature04754

[27] Acosta-Rodriguez EV, Napolitani G, Lanzavecchia A, Sallusto F (2007) Interleukins 1β and 6 but not transforming growth factor-β are essential for the differentiation of interleukin 17-producing human T helper cells. Nat Immunol 8: 942–949. 17676045 10.1038/ni1496

[28] Van de Veerdonk FL, Marijnissen RJ, Kullberg BJ, Koenen HJPM, Cheng SC, et al (2009) The macrophage mannose receptor induces IL-17 in response to *Candida albicans* . Cell Host Microbe 5: 329–340. 19380112 10.1016/j.chom.2009.02.006

[29] McGeachy MJ, Chen Y, Tato CM, Laurence A, Joyce-Shaikh B, et al (2009) The interleukin 23 receptor is essential for the terminal differentiation of interleukin 17-producing effector T helper cells in vivo. Nat Immunol 10: 314–324. 19182808 10.1038/ni.1698 PMC2945605

[30] Jiang Q, Li WQ, Aiello FB, Mazzucchelli R, Asefa B, et al (2005) Cell biology of IL-7, a key lymphotrophin. Cytokine Growth F R 16: 513–533. 10.1016/j.cytogfr.2005.05.004 15996891

[31] Kondrack RM, Harbertson J, Tan JT, McBreen ME, SurhCD, et al (2003) Interleukin 7 regulates the survival and generation of memory CD4 cells. J Exp Med 198: 1797–1806. 14662907 10.1084/jem.20030735 PMC2194153

[32] Osborne LC, Dhanji S, Snow JW, Priatel JJ, Ma MC, et al (2007) Impaired CD8 T cell memory and CD4 T cell primary responses in IL-7R alpha mutant mice. J Exp Med 204: 619–631. 17325202 10.1084/jem.20061871 PMC2137912

[33] Miossec P, Korn T, Kuchroo VK (2009) Interleukin-17 and type 17 helper T cells. New Engl J Med 361: 888–898. 19710487 10.1056/NEJMra0707449

[34] Jovanovic DV, Martel-Pelletier J, Di Battista JA, Mineau F, Jolicoeur FC, et al (2000) Stimulation of 92-kd gelatinase production by interleukin-17 in human monocyte/macrophages. Arthritis Rheum 43: 1134–1144. 10817568 10.1002/1529-0131(200005)43:5<1134::AID-ANR24>3.0.CO;2-#

[35] Rifas S, Arackal S (2003) T cells regulate the expression of matrix metalloproteinase in human osteoblasts via a dual mitogen-activated protein kinase mechanism. Arthritis Rheum 48: 993–1001. 12687541 10.1002/art.10872

[36] Hirota K, Yoshitomi H, Hashimoto M, Maeda S, Teradaira S, et al (2007) Preferential recruitment of CCR6-expressing Th17 cells to inflamed joints via CCL20 in rheumatoid arthritis and its animal model.J Exp Med. 204: 2803–2812. 10.1084/jem.20071397 PMC2118525 18025126

[37] Wang C, Kang SG, Lee J, Sun Z, Jim CH (2009) The roles of CCR6 in migration of Th17 cells and regulation of effector T-cell balance in the gut. Mucosal Immunol 2: 173–183. 19129757 10.1038/mi.2008.84 PMC2709747

[38] Kantele A, Zivny J, Hakkinen M, Elson CO, Mestecky J (1999) Differential homing commitments of antigen-specific T cells after oral or parenteral immunization in humans. J Immunol 162: 5173–5177. 10227989

[39] Rott LS, Rose JR, Bass D, Williams MB, Greenberg HB, et al (1997) Expression of mucosal homing receptor alpha4beta7 by circulating CD4+ cells with memory for intestinal rotavirus. J Clin Invest 100: 1204–1208. 9276738 10.1172/JCI119633 PMC508297

[40] Tager AM, Bromley SK, Medoff BD, Islam SA, Bercury SD, et al (2003) Leukotriene B4 receptor BLT1 mediates early effector T cell recruitment. Nat Immunol 4: 982–990. 12949531 10.1038/ni970

[41] Luster AD, Alon R, von Andrian UH (2005) Immune cell migration in inflammation: present and future therapeutic targets. Nat Immunol 6: 1182–1190. 16369557 10.1038/ni1275

[42] Bettelli E, Korn T, Kuchroo VK (2008) Induction and effector functions of TH17 cells. Nature 453: 1051–1057. 18563156 10.1038/nature07036 PMC6280661

[43] Happel KI, Dubin PJ, Zheng M, Ghilardi N, Lockhart C, et al (2005) Divergent roles of IL-23 and IL-12 in host defense against *Klebsiella pneumoniae* . J Exp Med 202: 761–769. 16157683 10.1084/jem.20050193 PMC2212952

[44] Ye P, Rodriguez FH, Kanaly S, Stocking KL, Schurr J, et al (2001) Requirement of interleukin 17 receptor signaling for lung CXC chemokine and granulocyte colony- stimulating factor expression, neutrophil recruitment, and host defense. J Exp Med 194: 519–527. 11514607 10.1084/jem.194.4.519 PMC2193502

[45] Higgins SC, Jarnicki AG, Lavelle EC, Mills KH (2006) TLR4 mediates vaccine-induced protective cellular immunity to *Bordetella pertussis*: role of IL-17-producing T cells. J Immunol 177: 7980–7989. 17114471 10.4049/jimmunol.177.11.7980

[46] Malley R, Srivastava A, Lipsitch M, Thompson CM, Watkins C, et al (2006) Antibody- independent, interleukin-17A-mediated, cross-serotype immunity to pneumococci in mice immunized intranasally with the cell wall polysaccharide. Infect Immun 74: 2187–2195. 16552049 10.1128/IAI.74.4.2187-2195.2006 PMC1418935

[47] Staal FJ, Luis TC, Tiemessen MM (2008) WNT signalling in the immune system: WNT is spreading its wings. Nat Rev Immunol 8: 581–593. 18617885 10.1038/nri2360

[48] Yu Q, Sharma A, Oh SY, Moon HG, Hossain MZ, et al (2009) T cell factor 1 initiates the T helper type 2 fate by inducing the transcription factor GATA-3 and repressing interferon-gamma. Nat Immunol 10: 992–999. 19648923 10.1038/ni.1762 PMC2824257

[49] Notani D, Gottimukkala KP, Jayani RS, Limaye AS, Damle MV, et al (2008) Global regulator SATB1 recruits beta-catenin and regulates TH2 differentiation in Wnt-dependent manner. PLoS Biol. 8: e1000296. 10.1371/journal.pbio.1000296 PMC2811152 20126258

[50] Yu Q, Sharma A, Ghosh A, Sen JM (2011) T cell factor-1 negatively regulates expression of IL-17 family of cytokines and protects mice from experimental autoimmune encephalomyelitis. J Immunol 186: 3946–3952. 21339363 10.4049/jimmunol.1003497 PMC3158594

[51] Stockinger B, Veldhoen M, Martin B (2007) Th17 T cells: linking innate and adaptive immunity. Semin Immunol 19: 353–361. 18023589 10.1016/j.smim.2007.10.008

[52] Kim J, Kim DW, Ghosh A, Sen JM (2012) Wnt5a is secreted by follicular dendritic cells to protect germinal center B cells via Wnt/Ca^2+^/NFAT/NF-κB-B Cell lymphoma 6 signaling. J Immunol 188: 182–189. 22124122 10.4049/jimmunol.1102297

[53] Lowrey JA, Stewart GA, Lindey S, Hoyne GF, Dallman MJ, et al (2002) Sonic hedgehog promotes cell cycle progression in activated peripheral CD4^+^ T lymphocytes. J Immunol 169: 1869–1875. 12165511 10.4049/jimmunol.169.4.1869

[54] Stewart GA, Lowrey JA, Wakelin SJ, Fitch PM, Lindey S, et al (2002) Sonic hedgehog signaling modulates activation of and cytokine production by human peripheral CD4^+^ T cells. J Immunol 169: 5451–5457. 12421920 10.4049/jimmunol.169.10.5451

[55] Sacedon R, Diez B, Nunez V, Hernandez-Lopez C, Gutierrez-Frias C, et al (2005) Sonic hedgehog is produced by follicular dendritic cells and protects germinal center B cells from apoptosis. J Immunol 174: 1456–1461. 15661904 10.4049/jimmunol.174.3.1456

